# Observing and treating pain in people living with dementia in long-term care facilities

**DOI:** 10.3389/fpain.2026.1812648

**Published:** 2026-05-08

**Authors:** Sabine D. Kruijer, Wilco P. Achterberg, Annelore van Dalen-Kok, Monique A. A. Caljouw

**Affiliations:** 1 Argos Zorggroep, Schiedam, Netherlands; 2 Department of Public Health and Primary Care, Leiden University Medical Center, Leiden, Netherlands; 3 University Network for the Care Sector South Holland, Leiden University Medical Center, Leiden, Netherlands; 4 Stichting Florence, The Hague, Netherlands

**Keywords:** dementia, long-term care, non-pharmacological interventions, pain, pain assessment, pain management, pharmacological interventions

## Abstract

**Objectives:**

Neuropathological changes in dementia compromise communicative abilities, causing pain to present atypically and making assessment challenging. Although pain is highly prevalent in people with dementia and can be managed pharmacologically and non-pharmacologically, comprehensive studies on pain assessment and management in this population remain scarce. This study aimed to explore how pain is observed, assessed, and managed in people living with dementia in long-term care facilities.

**Design:**

Mixed-methods sequential explanatory design.

**Setting and participants:**

Medical staff, healthcare professionals, nursing staff, and managers employed in long-term care facilities, as well as informal caregivers of residents with dementia in these facilities, in the Netherlands.

**Methods:**

Between April 9 and July 1, 2024, participants completed a nationwide survey (*N* = 387) containing questions about pain observations and the use of non-pharmacological and pharmacological interventions. The number of questions varied from 4 (for the managers) to 15 (for informal caregivers), and additional in-depth interviews were conducted (*N* = 20). Quantitative data were analyzed descriptively using SPSS to calculate frequencies and distributions. The interview summaries were analyzed for recurring themes and illustrative quotes used to support survey findings.

**Results:**

Pain was most considered when medical and nursing staff observed non-verbal indicators such as behavioral changes, supported by physical examination and, in some cases, pain observation scales. Approximately half of respondents reported using observational pain scales in routine practice. Pain management relied predominantly on pharmacological treatment, primarily paracetamol, with less frequent and unsystematic use of non-pharmacological interventions such as distraction, exercise, and music therapy.

**Conclusion:**

Pain assessment and management for people with dementia in long-term care facilities remain predominantly pharmacological. The limited and inconsistent use of validated observational tools and non-pharmacological interventions highlights the need for systematic implementation in clinical practice and future research.

## 1 Introduction

Half of the people living with dementia in long-term care facilities experience pain regularly (1). Despite this high prevalence, pain in people with dementia is often not recognized and therefore undermanaged (2). One of the main reasons for this is the loss of communicative abilities, as people with dementia are frequently unable to verbally express their pain effectively (3). As a result, pain may manifest indirectly through challenging behaviors such as agitation, aggression, and depression, as well as changes in physical functioning (4). The range of pain-related behaviours can also include observable indicators such as body gestures, facial expressions, and vocalizations, which are commonly used in observational pain assessment in dementia (5). Together, these behavioural and physical changes may signal the presence of pain (6–10).

Given these communication difficulties, observational tools are essential for assessing pain in people with dementia who cannot communicate verbally (11). These tools focus on pain-related behaviors such as facial expressions, movement, and vocalization (12). Although research shows that systematic use of observational tools can improve pain management, their implementation in daily practice remains inconsistent (2). Previous research shows that changes in behavior or physical functioning may indicate the presence of pain in people with dementia (13). Informal caregivers, who know the person with dementia well, are particularly able to recognize subtle changes in behavior or functioning, thereby contributing to more accurate pain identification.

In terms of treatment, medication remains the primary approach to pain management in people with dementia (2). Nevertheless, several guidelines recommend non-pharmacological interventions before initiating pharmacological treatment, including the guideline followed by elderly care physicians working in Dutch nursing homes (Verenso) and best-practice guidance for health professionals in the United Kingdom (14).

These recommendations reflect the limitations of pharmacological interventions in older persons (15). Analgesics can cause adverse effects in people with dementia, such as drowsiness and an increased risk of falling. For this reason, non-pharmacological interventions are often preferred as a first step for treating mild pain, while pharmacological treatment may be added for moderate to severe pain to achieve a synergistic effect (15).

In this context, non-pharmacological interventions—defined as “therapies that do not involve taking medicine or any other active substance” (16)—have gained increasing attention. Evidence from older adults without dementia supports interventions such as acupuncture, mindfulness, meditation, massage, and cognitive behavioral therapy (17). More recently, systematic reviews and meta-analyses in people with dementia indicate moderate efficacy for sensory-based interventions, such as reflexology and ear acupressure, and more consistent effects for interactive interventions, including music, singing, art-based activities, play, and robotic care (4, 18–20). However, effectiveness varies by intervention type, frequency, and care setting. A summary of these findings is provided in Table 1.

**Table 1:** Summary of a literature review on the effectiveness of non-pharmacological interventions in pain in dementia (in nursing homes).

Author, year	Study design	Setting	Diagnosis	Interventions	Results
Bao & Landers (18)	Mixed methods systematic review 8 studies	Residential care settings, memory clinics, patients' homes.	People with dementia Diagnosed with pain using SVS, PAINAD, M-PADE, DOLOPLUS	**Interactive interventions:**	
				Singing & painting:	Duration of pain decreased significantly from baseline compared to week 16 (*p* = 0.01). Intensity of pain was significantly reduced from baseline to week 16 (*p* = 0.009). Overall pain levels decreased but did not reach a significant level. Both interventions are equally effective at reducing pain.
				Play activities:	Reduction of pain intensity (*d* = 2.47).
				Therapeutic: robot	Patients calmed down resulting in reduction of pain.
				**Non-interactive interventions:**	
				Massage:	one study showed no statistical significance (*p* = 0.93) in the reduction of pain. Another study showed statistical significance pain reduction (*p* *<* 0.001)
				Listening to music:	No significant reduction of pain (*p* = 0.0582) but it can distract from the pain.
				Ear acupressure:	Was significant in effectively managing pain (*p* < 0.001)
Pu et al. (20)	Systematic review of randomized controlled trials 9 articles on 8 studies RCT design (parallel, cross-over, cluster, multi-arm, cluster-study with multi-arm)	Long-term care facility, the community, and a memory clinic	Dementia or MCI Pain measured with self-report (NRS, SVS, VDS) or pain observation scales (PACSLAC, CNPI, PAINAD, BPI, DOLOPLUS 2, DS-DAT, WOMAC)	Sensory stimulation: *(reflexology, massage, ear acupressure, music, showering)*	Statistically significant reduction of observed pain (SMD = −0.58, 95% CI [ −0.99, −0.17)
				Physical activity intervention: *(Tai Chi, Passive movement therapy)*	No statistical significance in reduction of pain (SMD = −0.24, 95% CI [ −1.06,0.59)
				Individual intervention:	Significant result in reduction of pain (SMD = −0.55, 95% CI [ −1.01, −0.09)
				Group interventions:	No significant reduction in pain (SMD=−0.27, 95% CI [ −1.06, 0.53)
				Medication use:	No significant effect was found, but a decrease in daily acetaminophen dosage was found for the Tai Chi group, compared to an increase in the control group.
Liao et al. (19)	Systematic review 11 articles Quantitative research	Community-dwelling individuals, hospital settings, long-term care facilities	Dementia and no dementia (separate results for people with dementia and people without dementia) Dementia diagnoses Pain: persons experiencing pain, chronic pain, or people taking pain medication for at least one month Pain assessment: PAINAD, M-PADE, DOLOPLUS 2, BPI, Algoplus Tool, CNPI, GMPI, Abbey pain scale, NRS and SVS	Massage:	One of the studies reported no significant change in self- reported pain in the intervention group (*p* = 0.21). Another study reported a decrease in pain (lower back pain and limb pain) post-intervention. Pain increased slightly after two months. The control group showed an increase in pain after regular care and an increase in pain after follow-up.
				Ear acupressure:	Significant reduction in pain measured after two months (*p* = 0.001)
				Music therapy:	Significant reduction in pain when listening to preferred music for more than 30 min (*p* < 0.05).
				Painting and singing:	Pain scored with SVS and BPI; significant reduction in pain after the intervention (*p* = 0.01, *p* = 0.009, respectively). When measured with the NRS, no significance reduction in pain (*p* = 0.057).
				Exercise:	No significant decrease in pain.
				Social activities:	No significant decrease in pain.
				Cognitive- Behavioral therapy:	Significant reduction in pain scores (*p* < 0.001).
				Personal assistive robot (PARO):	Significant pain reductions when measured with the PAINAD (*p* = 0.004), no significant reduction when measured with the NRS. Medication use was significantly lower in the intervention group (*p* = 0.025).
				Reflexology:	Significantly reduction in pain scores after intervention.
				Tailored pain intervention:	No significant reduction in pain scores.
				Play activity:	Significant reduction in chronic pain (*p* *=* 0.019) and significant difference in pain reduction between the intervention group and control group (*p* = 0.002.
				Patient- centered environmental program:	Significant reduction in pain severity after intervention (*p* = 0.002).
Saragih et al. (4)	Meta-analysis and a systematic review RCT, quasi-experimental study or experimental study	Nursing home, elder care facility, memory care facility, community setting	People with dementia and mild to moderate MCI Pain measured with PAINAD, C-PAINAD, PACSLAC-D, CNPI, VAS, AQoL-8D, NRS, and VDS	Massage, exercise, meditation, painting instruction and robotic care	**Overall effects assessed immediately after the intervention** No significant reduction in pain between the intervention and the control group. **Subgroup analyses** Intervention delivery frequency: Significant reduction in pain in the two intervention groups (interventions given once or twice a week and interventions given three times or more a week) (*p* *=* *.86, p* = .85, respectively). Post-intervention evaluations: Significant pain reduction 4–8 weeks after the intervention, compared with the control group. Results were greater after 4–8 weeks then diminished after more than 8 weeks.

DSM-V, Diagnostic and Statistical Manual of Mental Disorders fifth edition; NINCDS, National Institute of Neurological and Communicative Disorders and Stroke; ADRDA, Alzheimer's Disease and Related Disorders Association; SVS, Simple Visual Scale; PAINAD, Pain Assessment in Advanced Dementia; M-PADE, Modified Pain Assessment in the Dementia Elderly; MCI, Mild Cognitive Impairment; RCT, Randomized Controlled Trial; NRS, Numeric Rating Scale; VDS, Verbal Descriptor Scale; PACSLAC, Pain Assessment Checklist for Seniors with Severe Dementia; CNPI, Checklist of Nonverbal Pain Indicators; BPI, Brief Pain Inventory; DS-DAT, Discomfort Scale of Dementia of the Alzheimer's Type; WOMAC, Western Ontario and McMaster Universities Osteoarthritis Index; FAST, Functional Assessment Staging Scale; MMSE, Mini-Mental State Examination; GMPI, Geriatric Multidimensional Pain and Illness Inventory; VAS, Visual Analogue Scale; AQoL-8D, Assessment Quality of Life-8D.

Although pain in dementia is complex and increasingly prevalent due to demographic ageing, research describing the daily practices of observing and managing pain remains scarce. In particular, the use of systematic observational pain assessment tools and how different caregiver groups—such as medical staff, nursing staff, management, and informal caregivers—are involved in pain management (2, 21, 22).

The Dutch long-term care setting provides a relevant context to study these issues, as care for people with advanced dementia is highly organized, multidisciplinary, and guided by national recommendations for pain management (23). At the same time, variability in implementation across facilities may limit optimal pain care. Understanding usual care in this context is therefore relevant not only nationally, but also internationally, as many countries face similar challenges in translating evidence-based pain management strategies into routine long-term dementia care (24). Therefore, this study aims to provide an overview of how pain is recognized, assessed, and managed in long-term care facilities, with specific attention to the roles and perspectives of different caregiver groups. Also, given their growing role in dementia care in the Netherlands, this study aimed to explore whether informal caregivers are currently involved in assessing pain in people with dementia and applying non-pharmacological interventions.

## 2 Methods

### 2.1 Study design

A mixed-methods sequential explanatory design was used, consisting of an online survey and in-depth interviews. The study was exempt from review by the Medical Ethics Review Committee, as the non-WMO Review Committee determined that the Dutch Medical Research Involving Human Subjects Act (WMO) did not apply (protocol nr. N 24-3008).

### 2.2 Recruitment

Participants were recruited via academic care networks (“University Network for the Care sector South- Holland” (UNC-ZH) (25), “Collaborating Academic Networks for the Elderly Care” (Samenwerkende Academische Netwerken Ouderenzorg or SANO). Through SANO regional academic research networks were approached like the Academic Networks of Elderly Care in the North (UNO-UMCG) and the University Network for Elderly Care in North-Holland (UNO Amsterdam) to distribute the survey in several long-term care facilities in the northern part of the Netherlands. Recruitment was also done through social media and by the UNC-ZH website. Informal caregivers were approached through Minters (36), which distributed flyers during educational meetings and Alzheimer Nederland (a network for people with dementia and their loved ones).

### 2.3 Sample size

A total of 220 respondents were expected to be representative of the population: 150 medical staff, healthcare professionals, and nursing staff; 20 nursing home managers; and 50 informal caregivers (26). No formal power calculation was conducted. The obtained sample size is considered to provide a reasonable representation of the different professional groups.

### 2.4 Survey

An online survey was developed by (SK and MC) based on clinical experience and recent literature. The purpose of this survey was to gather information from different caregiver groups about the assessment and observation of pain, its treatment, and the roles these groups have within the overall process of pain management. They survey was validated with the LUMC Validation Castor Checklist, where the first and second part of the checklist was filled out by the researchers and the third part was filled out by an independent party. Non-pharmacological interventions included in the survey were derived from a search for articles on systematic reviews and meta-analyses conducted in 2023, using the following search criteria: “Pain” AND “Dementia” AND “Non-pharmacological interventions”, and published after 2020, to obtain most recent researched interventions (4, 18–20). Interventions shown to reduce pain in people with dementia were included (Table 1).

After providing informed consent, eligible participants answered demographic questions and were directed to a role-specific survey based on their relationship with a person with dementia living in a long-term care facility (medical staff, healthcare/nursing staff, management, or informal caregivers). Surveys differed by role to reflect variations in responsibility and involvement in pain recognition, assessment, and management. Most questions were multiple choice, with occasional free-text options. Participants received a notification when a question was not answered to ensure that all questions were answered and no data was missing. Participants who did not meet the inclusion criteria were unable to access the survey. The full survey is shown in Table 2. At the end of the survey, participants could indicate interest in an in-depth interview.

**Table 2:** Questions in the survey and results perrelationship.

Questions			Medical staff (*N* = 98)		Healthcare professionals (*N* = 131)		Nursing staff (*N* = 81)		Informal caregivers (*N* = 53)		Managers (*N* = 24)	
			*n*	%	*n*	%	*n*	%	*n*	%	*n*	%
Assessing pain												
*1. When is pain considered?*^a^ *(y)*												
Change in behavior			98	100	125	95.4	80	100.0				
Lab results			16	16.3	44	33.6	24	30.0				
Completing pain observation checklists every so often			23	23.5	97	74.0	58	72.5				
Observations from nursing staff			96	98.0	–	–	–	–				
Observations from family members			95	96.9	108	82.4	53	66.3				
No involvement in pain assessment			–	–	8	6.1	0	0				
Other (non-verbal expressions, after trauma, decreased functionality, mood swings, self-reports of pain, change in sleep patterns)			15	15.3	17	13.0	4	5.0				
Missing (NA, NASK)							1					
*2. Are you informed whether your family member is in pain?*^b^ *(y)*									44	83.0		
*2.1. Are you informed about whether the cause is being investigated?(y)*									32	60.4		
Missing (NA, NASK)									10			
*3. How is pain diagnosed?*^a^ *(y)*												
Physical examination			89	90.8	2	1.7	0					
Pain observation checklists			81	82.7	1	0.9	0					
Observations by the physician			94	95.9	2	1.7	0					
Starting trial medication			74	75.5	–	–	0					
Other (consultation with informal caregivers, observations during ADL, observations of changes in posture)			4	4.1	0		0					
Missing (NA, NASK)			1		15		13					
*4. Have you ever been asked to observe pain?*^b^ *(y)*					94	72.3	72	88.9	12	22.6		
Missing (NA, NASK)									1			
*4.1. Do you use a pain observation checklist for this?(y)*	115	54.2			59	62.8	56	78.9				
	47				37		10					
Missing (NA, NASK)												
*5. Have you ever reported that your family member is in pain? (y)*									43	81.1		
Missing (NA, NASK)									1			
*6. What follow-up actions are taken?*^a^ *(y)*												
Start pain medication			82	83.7								
Gather more information			97	99								
Consult with care staff and other disciplines			86	87.8								
Consult with family			68									
Other (SI investigation, involving other disciplines, consultation with the patient, using non-pharmacological interventions)			7	7.1								
Missing (NA, NASK)			1									
*7. Is family ever asked to observe pain? (y)*			40	40.8								
Missing (NA, NASK)			1									
Management of pain												
*8. Is pain always treated? (y)*			77	78.6								
Missing (NA, NASK)			1									
*9. Are you involved in creating a treatment plan?*^b^ *(y)*					89	69.0	54	68.4	2	3.8	4	16.7
Missing (NA, NASK)					2		2		43			
*9.1. Involvement by: (y)*												
Managing the team											4	16.7
Setting goals											1	4.2
Brainstorming treatment options											3	12.5
Considering possible interventions											2	8.3
Evaluating whether a resident is suitably placed in the specific long-term care facility											3	12.5
Other ways											–	–
Missing (NA, NASK)											20	
*10. Are guidelines followed? (y)*											23	95.8
Missing (NA, NASK)											1	
*10.1. Are you informed of deviations from the guidelines? (y)*											11	45.8
Missing (NA, NASK)											2	
*11. What is the basis for the treatment plan?*^a^ *(y)*												
General guidelines			66	67.3								
Own guidelines			16	16.3								
Own knowledge			55	56.1								
Other (palliative care, self-care products used)			2	2.0								
Missing (NA, NASK)			23									
*12. Do you have any non-pharmacological interventions at your disposal?(y)*			87	88.8								
Missing (NA, NASK)			3									
*13. Are you familiar with non-pharmacological interventions?*^b^ *(y)*									30	56.6		
Missing (NA. NASK)									1			
*13.1. Which of the following non-pharmacological interventions do you know?*^a^ *(y)*												
Music therapy					101	79.5	59	73.8	18	34.0		
Massage					96	75.6	51	63.7	16	30.2		
Activities matching interests					109	85.8	57	71.3	18	34.0		
Exercise					114	89.8	67	83.8	21	39.6		
Painting					67	52.8	29	36.3	6	11.3		
Acupressure					22	17.3	12	15.0	9	17.0		
Singing					83	65.4	52	65.0	12	22.6		
No interventions used					1	0.8	3	3.8	—	—		
Other (proper footwear, Qwiek.up, snoezelen, adjusting environment and posture, distraction, aromatherapy)					47	37.0	19	23.8	6	11.3		
Missing (NA, NASK)					4				23			
*13.2. Are these interventions ever used?* (y)					111	88.8	65	82.3				
Missing (NA, NASK)					6							
*13.3. Have you ever been asked to perform an intervention for pain treatment? (y)*					68	54.4	32	41.0				
Missing (NA, NASK)					6							
*13.4. Were positive effects observed? (y)*			70	71.4	87	41.0			8	15.1		
Missing (NA, NASK)			24		113				39			
*14. Are any non-pharmacological interventions included in the treatment plan?*^b^ *(y)*			74	75.5	79	90.8	41	78.8				
Missing (NA, NASK)			13		44							
*14.1. Non-pharmacological interventions are not included because:* ^a^ *(y)*												
Insufficient staff			4	4.1	3	6.8	1	3.1				
Lack of time			4	4.1	2	4.5	2	6.3				
Lack of knowledge			8	8.2	6	13.6	10	31.3				
Not the right staff for the interventions			–	–	0	0	2	6.3				
Not considered			8	8.2	4	9.1	10	31.3				
No clarity about execution and effect			6	6.1	2	4.5	2	6.3				
Other (Verenso guidelines, role of family)			2	2.0	1	2.3	0	0				
Missing (NA, NASK)			87		87		49					
*14.2. Which interventions are included in the treatment plan/ have you been asked to perform?*^a^ *(y)*												
Music therapy			49	50.0	15	21.7	12	38.7				
Massage			28	28.6	21	30.4	13	41.9				
Activities matching interests			63	64.3	26	37.7	17	54.8				
Exercise			65	66.3	39	56.5	18	58.1				
Painting			20	20.4	2	2.9	3	9.7				
Acupressure			1	1.0	2	2.9	1	3.2				
Singing			27	27.6	10	14.5	7	22.6				
No interventions used			1	1.0	0	0	0					
Other (complementary care, aromatherapy, referrals to occupational therapist or physiotherapist, PDL, SI, heat and cold, spiritual care)			27	27.6	26	37.7	7	22.6				
Missing (NA, NASK)			24		62							
*14.3. Did the treatment reduce pain?*					62	91.2	25	80.6				
Missing (NA, NASK)					63		50					
*14.4. Which intervention showed positive effects?*^a^ *(y)*												
Music therapy			39	39.8								
Massage			22	22.4								
Activities matching interests			45	45.9								
Exercise			56	57.1								
Painting			8	8.2								
Acupressure			1	1.0								
Singing			23	23.5								
No interventions showed positive effects			1	1.0								
Other (complementary care, aromatherapy, referrals to occupational therapist or physiotherapist, PDL, SI, heat and cold, spiritual care)			22	22.4								
Missing (NA, NASK)			24									
*14.5. How are the effects observed?*^a^ *(y)*												
Physical examination			16	16.3								
Observation lists			30	30.6								
Observations by doctors and care staff			66	67.3								
Observations by family member			47	48.0								
Observations after stopping intervention			20	20.4								
Missing (NA, NASK)			25									
*15. Do you ever mention that non-pharmacological interventions are available? (y)*					2	28.6	11	100				
Missing (NA, NASK)					124		70					
*16. Have non-pharmacological interventions ever been used with your family member? (y)*									14	26.4		
Missing (NA, NASK)									23			
*17. Would you want pain medication to be started for your family member? (y)*									50	94.3		
Missing (NA, NASK)									1			
*18. Would you want non-pharmacological interventions to be used for pain? (y)*									41	77.4		
Missing (NA, NASK)									1			
*19. What interventions did your family member use at home to reduce pain?*^a^ *(y)*												
Pain medication									41	77.4		
Walking									14	26.4		
Sports									8	15.1		
Massage									8	15.1		
Meditation									–	–		
Other (acceptance, acupuncture, distraction, conversation, going to a doctor)									8	15.1		
Missing (NA, NASK)									2			
*19.1. Would you want this to still be used now? (y)*									42	79.2		
Missing (NA, NASK)									2			
*19.2. Has the staff ever asked about these interventions? (y)*									13	24.5		
Missing (NA, NASK)									2			
*19.3. Do you ever undertake activities to reduce pain for your family member? (y)*												
Missing (NA, NASK)									25	47.2		
*20. How long would you use non-pharmacological interventions? (y)*												
1–5 days			16	16.3								
1–2 weeks			26	26.5								
3 weeks–1 month			27	27.6								
What is indicated in literature or guidelines			10	10.2								
Missing (NA, NASK)			19									
*21. Which do you choose? (y)*												
First medication			18	18.4								
First non-pharmacological			6	6.1								
Combination			59	60.2								
Missing (NA, NASK)			15									
*22. Are you ultimately responsible? (y)*			68	69.4								
Missing (NA, NASK)			9									

ADL, General Daily Activities (Algemene Dagelijkse Levensverichtingen); SI, Sensory Information processing; PDL, Passivities of Daily Living; NA, Not Answered; NASK, Not Asked.

a Multiple answers possible.

b Question answered with “yes” directed to the follow-up question.

(y) the results are the percentage of “yes” answers to the question.

### 2.5 Participants and eligibility

Participants included medical staff (*elderly care physician, physician in training)*, healthcare professionals *(physical therapist, music therapist, psychologist, massage therapist etc.*), nursing staff (*nurses and nursing aids*), management from Dutch long-term care facilities, and informal caregivers of residents with dementia. All participants were older than 18 years old, proficient in the Dutch language, and involved in caring for people with dementia who experience communication difficulties. Professionals were required to have at least 12 months' experience in long-term dementia care. Informal caregivers had to be family members of residents living in a long-term care facility for at least six months and be responsible for medical decision-making.

### 2.6 Procedure and quantitative analysis

The survey was conducted in CASTOR and open from April 9 to July 1, 2024, and was accessible via a link shared on the collaborating networks' websites and in newsletters. Information about the study, how data was collected and how the results of the study would be used was also provided (27).

**Figure 1:**
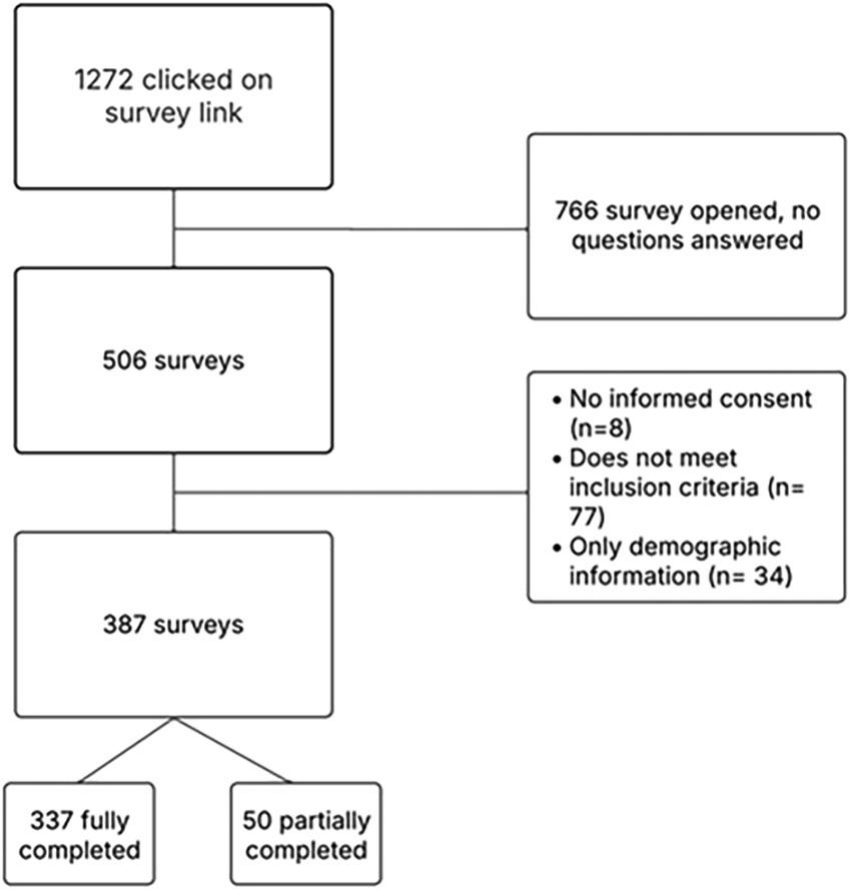
Summary of excluded surveys.

Surveys containing only demographic data were excluded from analysis (Figure 1). Results were analyzed using SPSS version 29. Quantitative analyses were primarily descriptive (Table 3). Frequencies, percentages, means, and standard deviations will be reported. Where appropriate, ranges are also provided.

**Table 3:** Demographic characteristics of the participants (*N* = 387).

Characteristics *n* (%)	
Age, *Mean* (SD)	48.5 (14.3)
Gender, *n* (%)	
Male	79 (20.4)
Female	305 (78.8)
Other	2 (0.5)
Missing (NA)	1 (0.3)
Highest level of education, *n* (%)	
Pre-vocational education (*VMBO*) or intermediate general secondary education (*MAVO*)	26 (6.7)
School of higher general secondary education (*HAVO*) or pre-university education (*VWO*)	63 (16.3)
Higher national education (*HBO-kort, HTI or HTS*) or Bachelor's degree	91 (23.5)
University Master's degree or PhD	205 (53.0)
Missing (NA)	2 (0.5)
Country of birth, *n* (%)	
Netherlands	364 (94.1)
Other (*Argentina, Belgium, Curacao, Ecuador, France, Germany, Hungary, Indonesia, Cameroon, Mauritius, Russia, Saint Maarten, Suriname, Syria, USA, South- Africa*)	23 (5.1)
Relationship with residents, *n* (%)	
Medical staff (elderly care physician, physician in training)	98 (25.3)
Healthcare professionals (physical therapist, music therapist, psychologist, massage therapist)^a^ and nursing staff (nurses and nursing aids)	212 (54.8)
Managers of a LTCF	24 (6.2)
Informal caregivers	53 (13.7)

Not Answered (NA), Long Term Care Facility (LTCF).

a One elderly care physician filled out the survey intended for healthcare professionals and nursing staff.

### 2.7 Interviews

Participants who were interested in attending in-depth interviews provided contact details at the end of the survey. Interviewees were purposively selected to ensure multidisciplinary representation and diversity based on gender, level of education and relationship to residents of the long-term care facilities. To obtain a divers representation of the participants, at least 15 interviews will suffice to support the results of the survey for more clarification.

### 2.8 Procedure and qualitative analysis

Participants were contacted via email first with additional information about the interview and the study. Then one of the researchers (SK or JL) contacted them by phone to schedule an interview appointment. The informed consent form was emailed and asked to sign the form before the interview started.

Interviews were semi-structured, conducted by (SK, Psychologist and PhD candidate or JL, Master student Psychology) via Microsoft Teams or at the participant's workplace, and focused on pain recognition, assessment, and management (Appendix 1). Interviews were recorded, transcribed verbatim, and thematically summarized. Each interview was transcribed by one researcher and subsequently checked for accuracy by the second researcher. Two researchers independently analyzed the data, and illustrative quotes were selected to complement or contrast survey findings.

The answers on the survey were analyzed and reported. Subsequently, two researchers independently reviewed the interview transcripts to identify quotes and information that either supported or contradicted the survey findings. These quotes and the information were then discussed collaboratively, and consensus was reached regarding the selection of the most representative and relevant quotes or information.

No formal codebook was developed. The primary analysis focused on the survey data. The transcribed interviews were subsequently reviewed to identify quotes that could support or contextualize the survey findings. The interviews were therefore used to complement and further illustrate the quantitative results rather than to conduct a separate qualitative analysis.

## 3 Results

### 3.1 Survey

A total of 1,272 potential participants clicked on the survey link (Figure 1). Of these, 766 opened the survey but did not answer any questions. 50 participants completed the survey partially, and 337 completed the entire survey. In total, 387 respondents who completed at least one substantive survey item, in addition to the demographic questions, were included in the quantitative analyses.

### 3.2 Interviews

At the end of the survey, 77 respondents indicated their willingness to participate in an in-depth interview. After being approached, 20 participants, participated in the interviews: four with medical staff and twelve with healthcare professionals and nursing staff. Only two informal caregivers and two managers were willing to participate in an interview. Given the study's primary focus on professional decision-making in pain assessment and management, informal caregivers were included to complement, rather than dominate, the qualitative findings.

### 3.3 Demographics

Survey participants had a mean age of 48.5 years (SD = 14.3; range 19–67), and 78.8% (*n* = 305) were female. More than half of the respondents (*n* = 205; 52.9%) had completed higher professional education or a university degree. Additional demographic characteristics of the survey sample are presented in Table 3.

Table 4 presents the professional distribution within the survey sample of healthcare professionals, which was subdivided into (1) nursing staff (registered nurses and nursing aids) and (2) healthcare professionals (e.g., physical therapists, psychologists, music therapists, and massage therapists).

**Table 4:** Professional groups within healthcare professionals and nursing staff and daily tasks^b^.

Professions	*N* (%)
Nursing staff	
Care assistant (*assist in personal care and help in daily activities*)	5 (6.2)
Nursing aids (*personal care, support in daily activities*)	28 (34.6)
Nurses (*direct care and medical procedures, like giving medication and wound care and guidance in daily life*)	48 (59.3)
Healthcare professional	
Psychologists (*diagnosing and treating psychiatric diseases*)	34 (8.8)
Physical therapists (*treatment, exercise therapy and prevention of falls*)	20 (5.2)
Occupational therapists (Music therapist, psychomotor therapist, creative therapist) (*treating and advising psychosocial and behavioural problems in residents with dementia*)	10 (2.6)
Occupational therapists (*assess what someone can still do independently and advising and training on the use of assistive devices*)	15 (3.9)
Social workers (*supporting residents with loss or coping and supporting family with feelings of guilt and acceptance*)	3 (0.8)
Employee activities and well-being (*organizing activities and giving individual attention*)	1 (0.3)
PA/nursing specialist (*diagnosing and treating geriatric diseases)* Others (Social workers, Employee activities and well-being, Social pedagogy, Livingroom assistants, Practical nurse/practical researcher, Professional mentor, Exercise therapist, Dementia case manager, Complementary care practitioner, Behavioral consultant, Spiritual Counselor, Quality practitioner, Speech therapist)	21 (5.4)
	27 (7.1)
Elderly care physician^a^ (*diagnosing and treating geriatric diseases*)	1 (0.3)

PA, Physician Assistant.

a One elderly care physician filled out the survey meant for health care professionals and nursing staff.

b Small differences in daily tasks can be seen in different long-term care organizations.

One elderly care physician completed the survey intended for healthcare professionals. The data from this participant was retained for overall descriptive statistics where the professional role was not a determining factor.

### 3.4 Recognizing and assessing pain

Among survey respondents, all medical staff (100%), health care professionals (95.4%) and nursing staff (100%) indicated that they consider pain when challenging behavior is observed. Observational pain scales were mentioned to assist in identifying pain by 23.5% of medical staff, 74.0% of healthcare professionals and 72.5% of nursing staff When asked to observe pain, 62.8% of the health care professionals and 78.9% of the nursing staff uses observational pain scales.

However, more discrepancy on this was illuminated by interview data. Medical staff as well as healthcare professionals and nursing staff described observational scales as useful for identifying pain and evaluating treatment effects, particularly when pain signals are unclear. At the same time, they emphasized that high workload and time pressure often lead nursing staff to draw rapid conclusions without extensive analysis of underlying causes.

In the survey medical staff reported that pain assessments were frequently informed by family members (96.9%). However, only 40.8% indicated that informal caregivers were actively asked to observe or formally assess pain using an observation scale. Informal caregivers confirmed that they often observe and report pain-related behaviors (81.1%), yet only 22.6% reported being explicitly asked to do so.

Interview participants across professional groups described nonverbal cues—such as grimacing, resistance during care, pushing staff away, agitation, or aggression—as key indicators of pain. One psychologist stated: “*Pain is often signaled through behavioral changes like agitation, aggression, or repetitive behavior.”* Additional survey results on pain recognition and assessment are presented in Table 2.

### 3.5 Management of pain

Following pain assessment, medical staff reported several initial management steps: administering medication (83.0%), collecting additional information (99.0%), and consulting other disciplines (87.8%). Most medical staff (88.0%) indicated that non-pharmacological interventions were available to them and could be included in management plans. Nevertheless, a combination of medication and non-pharmacological interventions was included in only 60.2% of reported management plans.

Informal caregivers expressed a preference for combined approaches, indicating that they would initiate medication (94.3%) as well as non-pharmacological strategies (77.4%) when pain was suspected. Nearly half (47.2%) reported engaging in activities with their loved one to reduce pain.

### 3.6 Non-pharmacological interventions

Survey data showed that commonly recommended non-pharmacological interventions included exercise or movement, activities tailored to personal interests, and music therapy. These were reported by medical staff (66.3%, 64.3% and 50.0% respectively), healthcare professionals (56.5%, 37.7% and 21.7% respectively) and nursing staff (58.1%, 54.8% and 38.7% respectively). Other interventions mentioned less frequently included massage, yoga, and structured daytime activities.

Barriers to the use of non-pharmacological interventions were reported mainly by nursing staff and healthcare professionals and included insufficient knowledge about available interventions (respectively 31.3% and 13.6%) and failure to consider non-pharmacological interventions during the formulation of management plans (respectively 31.3% and 9.1%%).

Interview data added important nuance to these findings. Medical staff acknowledged that medications—particularly opioids—are often used first, despite growing awareness of their adverse effects. One elderly care physician noted: “*non-pharmacological interventions are used less frequently than medication and are often not used systematically.”* Limited staffing, time constraints, and lack of resources were repeatedly mentioned as barriers to implementing non-pharmacological interventions.

Several interviewees also offered suggestions to overcome these barriers, including better staff training, clearer protocols for non-pharmacological interventions, improved interdisciplinary collaboration, and greater involvement of informal caregivers. Informal caregivers emphasized that non-pharmacological interventions could be valuable complements to medication if they were structurally embedded in care routines.

No inferential comparisons between professional groups were conducted, as the study was exploratory in nature and group sizes were unequal.

### 3.7 Responsibility and decision-making

Medical staff were reported to hold primary responsibility for pain management plans (69.4%), typically guided by clinical guidelines. Managers became involved when deviations from these guidelines were considered. Despite the acknowledged role of informal caregivers in recognizing pain, interviews revealed that they were rarely involved in composing management plans.

## 4 Discussion

The purpose of this study was to provide an overview of how pain is observed, assessed, and managed in people with dementia living in long-term care facilities in the Netherlands. Overall, medical staff, healthcare professionals, and nursing staff reported considering pain when people with dementia exhibit behavioral changes. Although observational scales can be useful for assessing pain, only half of the respondents used them, often due to time constraints and high workloads. Medication is predominantly used in pain management (2) but our study found that non-pharmacological interventions were used more often than expected. This is in line with the mostly used guideline in Dutch long-term care facilities, the guideline of the Dutch Association of Elderly Care Physicians—Verenso—on pain in people with dementia (28). Informal caregivers were reportedly rarely involved in observing and managing pain in their loved ones.

Although observational pain scales are recommended in national and international guidelines [Verenso (14, 28)], it is not used systematically in practice. This limited use is consistent with international literature and highlights a persistent gap between guideline recommendations and daily clinical practice. While time constraints and high workloads were frequently cited as barriers in this study, these factors alone do not fully explain the underuse of observational tools. Additional systemic barriers may include limited training in pain assessment, lack of routine integration of pain scales into care workflows, insufficient organizational support, and the perception that observational tools are time-consuming or add limited value (21, 24, 29, 30). Addressing these barriers likely requires organizational-level interventions, such as embedding pain assessment tools into electronic care records, aligning assessments with routine care moments, and reinforcing their importance through leadership and education (31).

Pain was most often recognized through behavioral changes, a finding consistent with the study by Tagliafico et al. (32), which described clusters of pain-related behaviors including facial expressions and psychomotor and psychosocial changes (32). However, reliance on behavioral cues alone may increase the risk of misinterpretation, as such behaviors may also be attributed to dementia-related symptoms rather than pain (33, 34). This further underscore the importance of structured and validated observational tools to support clinical decision-making.

Informal caregivers were rarely involved in pain observation and management, despite their familiarity with the person with dementia. Bullock et al. (13) emphasized that knowing a person's usual behavior is crucial for optimizing pain assessment (13). Greater involvement of informal caregivers aligns with international principles of person-centered care and may improve the accuracy and quality of pain assessment.

Pain management remained predominantly pharmacological, consistent with earlier findings (2). Although non-pharmacological interventions were reported more frequently than expected, they were not consistently included in pain management plans. While interventions such as exercise, massage, distraction, and music therapy were mentioned, the frequency and duration of use and effectiveness were not mentioned. Chen et al. (35) demonstrated that interventions such as music therapy can reduce pain and depression and are low-cost, noninvasive, and relatively easy to implement (35). However, even such interventions require time, staff engagement, and organizational support to be used consistently in long-term care facilities (21, 31).

Standardized pain management guidelines and stepwise protocols for pain assessment and management in long-term care facilities may contribute to reducing the underdiagnosis and undertreatment of pain in individuals with dementia. Structured approaches, such as the *Sta-op!* method support systematic observation and identification of pain-related behaviors. Using this method in combination with the guidelines on pain in people with dementia, such as the Verenso Guideline, may promote increased use of non-pharmacological interventions for pain management (28). To our knowledge, this is the first study to provide an overview of how pain is observed, assessed, and managed in Dutch long-term care facilities. Strengths of this study include mixed-methods sequential explanatory design and the use of in-depth interviews to contextualize survey findings. The nationwide distribution of the survey contributes to the generalizability of the findings. Also, findings like the emphasis on behavioral changes as key indicators of pain in individuals with impaired communication are likely relevant beyond the Dutch context and reflect issues faced in long-term care facilities internationally. However, several limitations should be acknowledged. Participants were not asked to specify their long-term care facility, limiting insight into organizational diversity. Therefore, there could be a bias where facilities with solidly embedded pain management programs participated in the study, while facilities with less embedded programs did not. Also, participant were not asked which specific observational pain scales were used in practice. This information could have provided insight into the usability of different instruments. The small number of managers and informal caregivers restricts generalizability for these groups. Another limitation is that different subgroups have different roles in the interprofessional team that provides the pain observations and management, but the subgroups were too small to analyze separately. Both survey and interview data may be subject to socially desirable responses, and the study title may have led to overreporting of non-pharmacological intervention use. Self-reported measures of pain are widely considered the gold standard, including in populations with dementia. However, in the present study, specific inquiries regarding the utilization of self-report instruments for pain assessment were not conducted. Consequently, it remains unclear whether self-reports are employed in individuals with dementia, the frequency of their use, or whether individuals are first asked about their pain experience prior to implementing observational assessment tools. Additionally, most participants were female and highly educated, which may limit representativeness.

## 5 Implications for future research and practice

Future research should focus on the development and validation of standardized and feasible observational pain assessment protocols for long-term care facilities, and implementation studies examining non-pharmacological interventions, including their frequency, duration, effectiveness, and resource requirements. Such studies should apply implementation frameworks that address staff training, workflow integration, interdisciplinary collaboration, and structured involvement of informal caregivers.

## 6 Conclusion

Despite the high awareness of the relationship between pain and challenging behavior, pain assessment practices remain inconsistent. There is limited use of validated observational tools and input from informal caregivers is underutilized. Pain management is predominantly pharmacological, even though the value of non-pharmacological interventions is increasingly recognized. However, these interventions are underused due to a lack of knowledge, time, and resources. Informal caregivers have expressed a willingness to support pain assessment and management, yet they are rarely involved in decision-making processes. These findings underline the need for a more systematic, interdisciplinary approach to pain management in dementia care. This approach should include better integration of non-pharmacological interventions, and greater involvement of formal and informal caregivers in developing pain management plans.

## Data Availability

The raw data supporting the conclusions of this article will be made available by the authors, without undue reservation.

## Appendix 1

**Topic list**

**Assessment of Pain**

- Use of measurement scales: Are these used, and if so, why; if not, why not?
- Changes in behavior due to pain: Are changes observed in the behavior of the residents, and how is this managed? Is pain considered as a cause?
- Overlap between behavior and pain: When there are changes in behavior, what is the primary focus (cause or treatment)?

**Pain management**

- Medication: Is it commonly used for pain, and is it given on trial or based on scientifically validated surveys?
- Non-pharmacological interventions: Are they used, and if so, why; if not, why not? Are they used first, or is medication used initially?
- Duration of non-pharmacological treatment (these treatments often take longer to show effects than medication).
- Opinion on non-pharmacological treatment: Do you think it is important to try this first and then use medication, or to use a combination? What do you think about the use of non-pharmacological interventions for people who might not have much longer to live?

**Communication**

- Treatment plan: Is there communication about it? Do you have a say in it? Are non-pharmacological interventions included in the plan?
- Duration and outcome of treatment: How long would you use the intervention? How are the outcomes tracked? Are they evaluated?
- Tapering off medication: If medication is used alongside non-pharmacological interventions, is the medication tapered off? Is anything used when tapering off medication?
